# An Association of Multiple Well Differentiated Liposarcomas, Lipomatous Tissue and Hereditary Retinoblastoma

**DOI:** 10.1080/13577140500229596

**Published:** 2005

**Authors:** J. K. O'Neill, C. A. Stone, P. Sarsfield, M. Smith, S. F. Smithson, D. Silver, V. S. Devaraj

**Affiliations:** Department of Plastic and Reconstructive Surgery Royal Devon and Exeter Hospital Barrack Road Exeter EX2 5DS UK

## Abstract

Well differentiated liposarcoma (atypical lipomatous tumour) is a low grade tumour, with no metastatic potential unless dedifferentiation supervenes. When superficial, it recurs locally only occasionally after marginal excision. We present a patient in whom bilateral childhood retinoblastoma was followed by later development of massive confluent areas of low grade liposarcoma and lipomatous tissue affecting the upper extremities and trunk. We discuss the role of mutations in the retinoblastoma gene (RB1) in linking these conditions and demonstrate the surgical management of an extremely unusual and challenging case.


*Sarcoma*, September/December 2005; 9(3/4): 151–156

Taylor & Francis

Taylor & Francis Group

**CASE REPORT**

### An association of multiple well differentiated liposarcomas, lipomatous tissue and hereditary retinoblastoma

J. K. O'NEILL, C. A. STONE, P. SARSFIELD, M. SMITH, S. F. SMITHSON, D. SILVER, & V. S. DEVARAJ

*Department of Plastic and Reconstructive Surgery, Royal Devon and Exeter Hospital, Barrack Road, Exeter EX2 5DS, UK*

**Abstract**
Well differentiated liposarcoma (atypical lipomatous tumour) is a low grade tumour, with no metastatic potential unless dedifferentiation supervenes. When superficial, it recurs locally only occasionally after marginal excision. We present a patient in whom bilateral childhood retinoblastoma was followed by later development of massive confluent areas of low grade liposarcoma and lipomatous tissue affecting the upper extremities and trunk. We discuss the role of mutations in the retinoblastoma gene (RB1) in linking these conditions and demonstrate the surgical management of an extremely unusual and challenging case.

**Keywords:** *Liposarcoma, retinoblastoma, lipoma, genetic*

### Case report

A 49-year-old man presented to us in January 2003 with a fatty pedunculated lesion in the left axilla. He had a past history of multiple similar lesions (some of which recurred after initial excision) over his shoulders, upper back and arms. Operations to excise them were undertaken in 1979, 1985, 1993 and 1997. Clinically the lesions removed were superficial, discrete and mobile. These lumps developed on a background of abnormally large fatty tissue over shoulders, back and anterior chest which began to develop and increase gradually in size over the past 6 years. On examination he was found to have an extraordinary appearance with an unusual fat distribution. His large shoulders and buffalo hump back were clearly out of proportion to the rest of his habitus (Figure 1). He weighed 19.5 stone and was 5 ft 7.5 inches tall.

The lesion arising from the left axilla was excised under general anaesthetic as a daycase in April 2003.

The patient had a history of bilateral retinoblastoma diagnosed at 21 months of age. In order to treat this, he received radiotherapy to the right eye and underwent enucleation of the left eye.

There was no known family history of retinoblastoma or other tumours.

Histologically, a spectrum is seen within the tumours: areas typical of well differentiated liposarcoma (atypical lipomatous tumours) merge imperceptibly towards the periphery with histologically normal adipose tissue. The tumours include areas of increased cellularity with occasional atypical cells particularly in relationship to fibrotic bands running through the lesions (Figure 2(a)). In addition there is much variability in adipocyte size and shape and very occasional cells consistent with lipoblasts are seen (Figure 2(b)).

MR imaging was performed in June 2003 to assess the extent of the remaining swellings in his shoulders neck and arms. His shoulders were too large to fit into the body coil of the local MRI Scanner (Siemens 1.0T Magnetron Impact, Erlangen, Germany) and therefore imaging was performed at a different hospital on an 'open MRI scanner' (Siemens 0.2T OR10 Magnatron Open Resistive, Erelangen, Germany) which does not have the same physical constraints. This allowed each shoulder to be studied separately and then the thorax was scanned with his arms raised out of

Correspondence: Miss Jennifer O'Neill, Department of Plastic Surgery, Royal Devon and Exeter Hospital, Barrack Road, Exeter EX2 5DS, UK. Tel: 44 1392 411611. Fax: 44 1392 402820. E-mail: jenniferoneill@hotmail.com

ISSN 1357–714X print/ISSN 1369–1643 online/05/(03-04)0151-6 © 2005 Taylor & Francis
DOI: 10.1080/13577140500229596

(a)

(b)

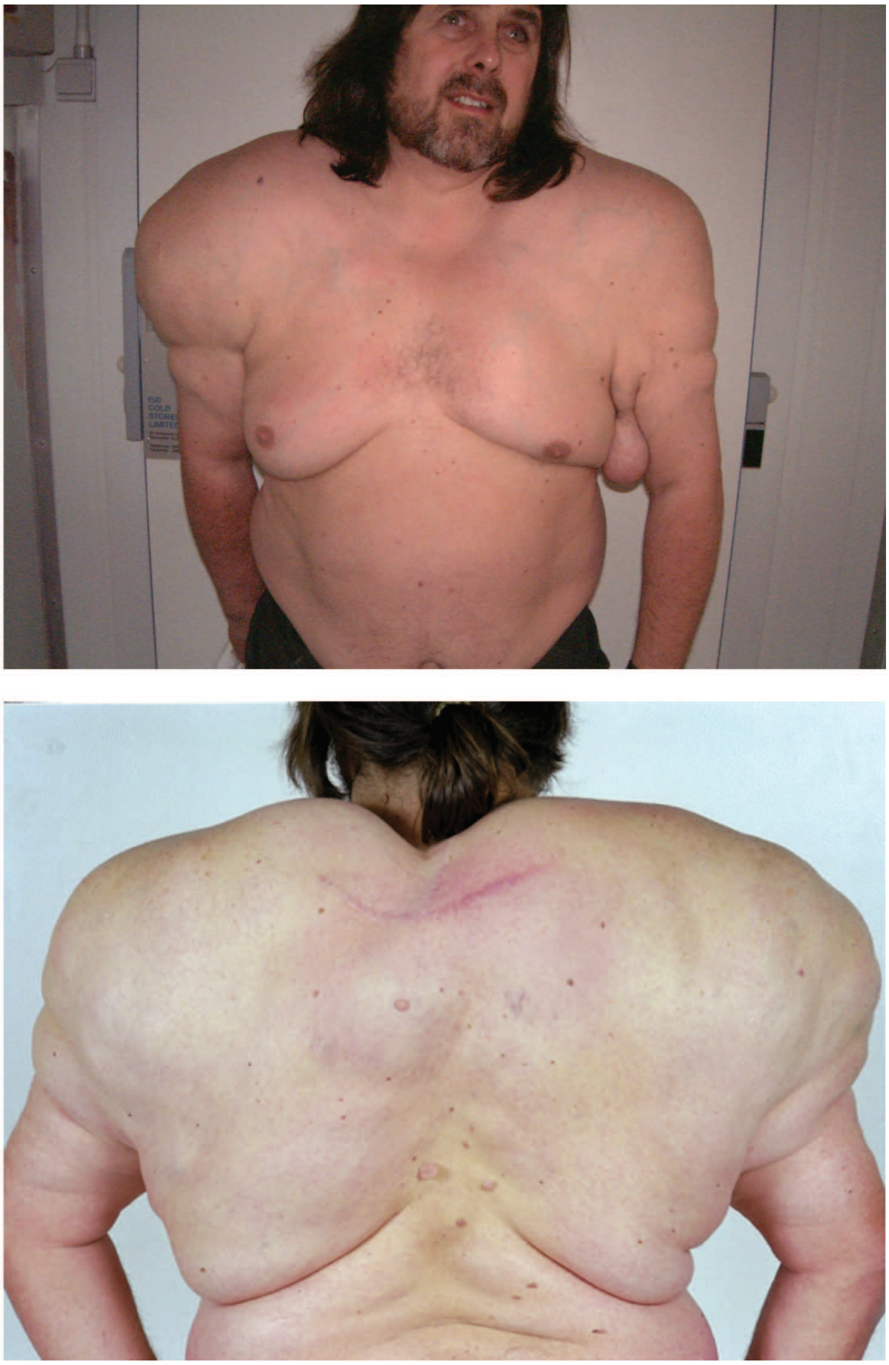

Figure 1. (a) Anterior view of abnormal body habitus and lipodystrophy. The fatty pedunculated lesion is visible in the left axilla. (b) Posterior view. A scar from previous surgery can be seen across the upper back.

the coil. Both shoulders showed marked fatty thickening in the subcutaneous tissues and this thickening passed along the arms and to the anterior chest wall. There was no evidence of abnormal fat deep to the muscle layers. Margins were not clearly defined between lesions and the whole mass of tissue appeared homogeneous. The STIR sequences best demonstrated the irregular nature of the swelling arising from the right axilla with a lobulated appearance and oedematous fluid within. T1 coronal images of both his shoulder girdles demonstrated the lobulated fatty soft tissue masses (Figure 3).

With regard to the liposarcomas, no further surgery or adjuvant therapy was planned. The patient was warned about the risk of dedifferentiation and continues on clinical follow up to check for any discrete, rapidly enlarging or painful nodules. Some further debulking to non-specific areas of lipodystrophy is currently being planned at the patient's request to aid mobility and cosmesis.

### Discussion

This is a case of a man with bilateral retinoblastomas, multiple liposarcomas (atypical lipomatous

(a)

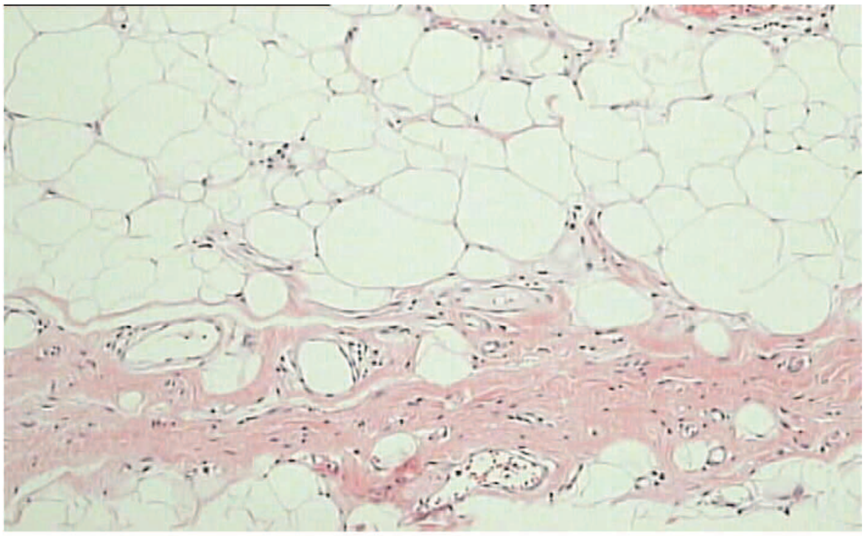

(b)

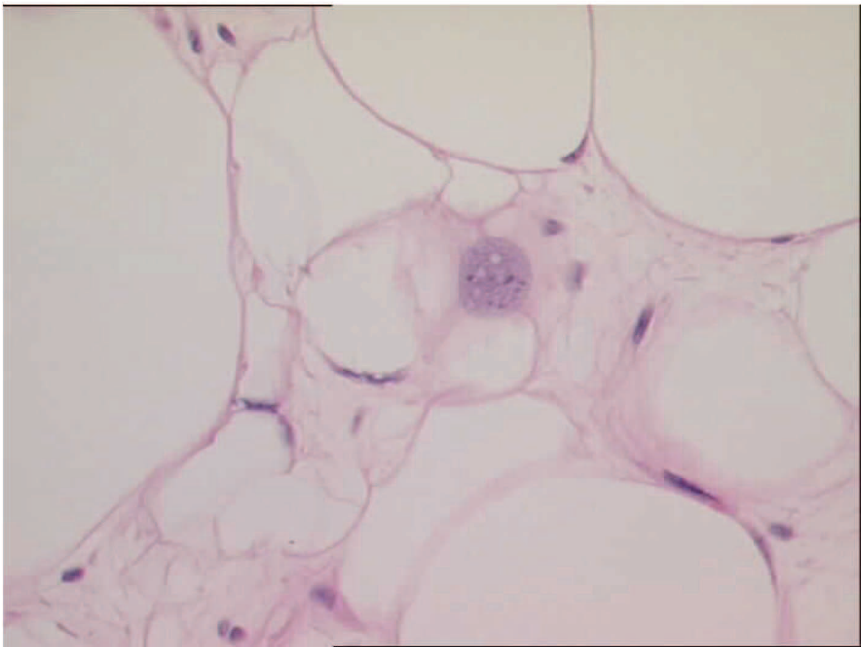

Figure 2. Photomicrographs of representative sections of the tumour stained with haematoxylin and eosin. (a) A fibrotic band is visible on this section. (b) A lipoblast cell is shown.

tumours), excess lipomatous tissue and an unusual body habitus. Retinoblastoma tumours are frequently caused by mutations of the RB1 tumour suppressor gene situated at chromosome 13q14.1 [1–3]. Most patients with bilateral disease have a germline (inherited) mutation of one copy of the gene. Tumours arise after a 'second hit' phenomenon producing a mutation on the other allele in retinal cells. Although there is no family history to suggest that previous generations of this man's family had the RB1 gene mutation, the presence of bilateral disease suggests that he has the familial form (whether inherited or occurring as a spontaneous new mutation) with a germline mutation affecting each cell.

The RB1 gene encodes a tumour suppressor protein which is present in all normal cell lines [1]. The RB1 gene protein actively regulates the cell cycle by acting as a brake in the advancement of cells from the G1 to the S phase of the cycle. It plays a critical role in determining cell cycle progression. If the RB1 protein is absent (due to mutations occurring in both copies of the gene) then progression into S phase occurs unchecked and cell proliferation is increased [1].

Retrospective cohort studies [4,5] have found that patients with bilateral retinoblastoma and, to a lesser extent unilateral retinoblastoma, have an increased risk of death from second primary neoplasms, particularly sarcomas of bone and connective tissue, skin melanomas and brain tumours. Radiotherapy as a primary treatment for retinoblastoma appears to further enhance the inborn susceptibility to develop a second cancer [4].

(a) (b)

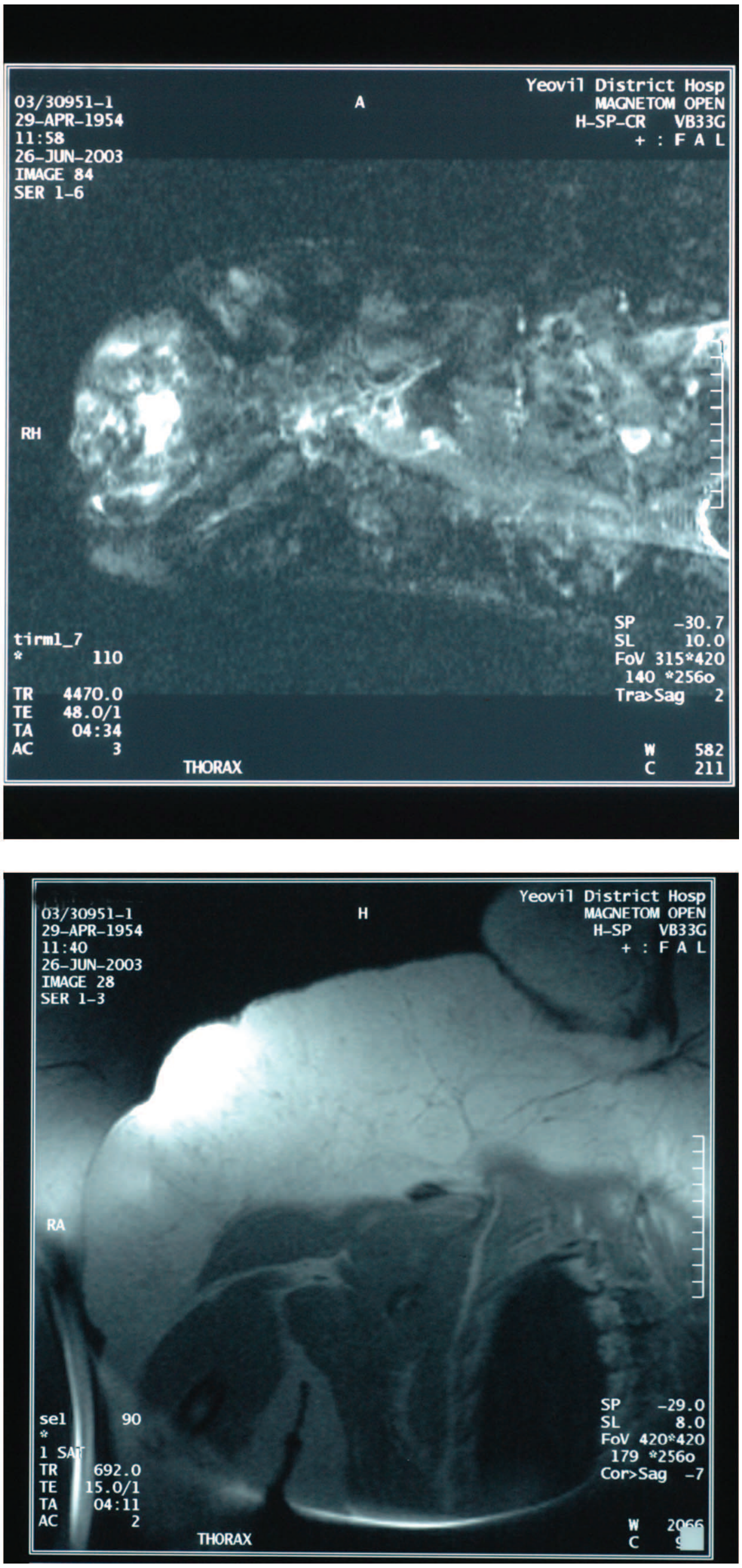

Figure 3. (a) STIR Axial image of right axilla demonstrating the irregular nature of the swelling arising from the right axilla. (b) Coronal T1 Image of the right shoulder demonstrating the lobulated fatty soft tissue masses arising in the subcutaneous fat over deltoid.

The rate of developing a second neoplasm after hereditary retinoblastoma is about 13% [6,7]. Two large series of patients with retinoblastoma have found previous cases of liposarcoma (one retroperitoneal) [7,8].

Deletions and structural rearrangements of regions on the long arm chromosome 13 near the RB1 locus have already been found to confer susceptibility to formation of benign and malignant lipomatous tumours (ordinary lipoma, spindle cell/pleomorphic lipoma, myxolipoma, angiomyxolipoma and liposarcoma) [2].

Multiple lipomas are reported to be associated with hereditary retinoblastoma [6,9,10]. Hereditary retinoblastoma patients with lipomas have a greater frequency (30%) of second malignant neoplasms (including malignant fibrous histiocytoma, leiomyosarcoma and fibrosarcoma) compared with patients in whom lipomas are not an associated feature (risk of approximately 13%) [6]. The sarcomas originated at sites separate from pre-existing lipomas, except for one patient with a scrotal leiomyosarcoma [6].

The lipomas associated with retinoblastoma may differ in presentation from other lipomatoses [10], such as familial multiple lipomatosis and benign cervical lipomatosis. RB-associated lipomas tend to be discrete and mobile lesions (as in familial multiple lipomatosis) and contrast with the diffuse fatty infiltration seen in benign cervical lipomatosis. Lipomas in patients with RB are distributed predominantly over the face, neck, shoulders and upper chest (as in benign cervical lipomatosis), but in familial multiple lipomatosis these areas are mostly spared. We report a more generalised abnormal upper body fat distribution with superadded discrete and mobile tumours.

Cytogenetic and molecular genetic evidence for the link between germline RB1 mutations and lipomas was initially revealed by FISH and microsatellite studies that showed that the DNA in the lipomas from patients with retinoblastoma had lost heterozygosity of the RB1 gene [9].

Molecular studies have also demonstrated an extended genetic family segregating a splice site mutation in the RB1 gene [10]. Almost all the adult carriers of this mutation have multiple lipomas with incomplete penetrance of retinoblastoma. However, this RB1 gene mutation has also been reported in a pedigree family in whom there were no lipomas [6]. This indicates that the lipoma predisposition in hereditary retinoblastoma may not be due to a specific RB1 gene mutation but be influenced by modifying factors linked to this gene. A specific allele of a modifying factor may cause lipoma predisposition in carriers of the oncogenic RB1 gene mutation.

There may be a similar genetic mechanism in this patient whereby the mutation in his RB1 gene makes him susceptible to a modifying factor predisposing to liposarcoma.

Alternatively there could be a 'second hit' (mutation of the second retinoblastoma gene) in already existing lipoma cells that causes them to develop into liposarcoma. This is the mechanism by which retina cells develop retinoblastoma.

The DNA from blood and tumour was harvested for study in our patient. So far, although a germline mutation has been found in the blood DNA, the tumour studies have not shown loss of heterozygosity. This implies that the genetic mechanism is likely to be susceptibility to a modifying factor rather than a 'second hit'.

With regards to the clinical management of this patient, clearance of the tumour is probably impossible because the margin of the tumour cannot be delineated radiologically. The probability of de-differentiation to a higher grade tumour is unlikely but possible. De-differentiated liposarcoma occurs most frequently (over 75% of cases) in intra-abdominal locations (most commonly in the retroperitoneum), deep soft tissues of the extremities (15%) and the trunk (7%), and is very rarely seen in subcutaneous adipose tissue [11–13]. In a series of 155 cases of liposarcoma that de-differentiated to high grade, three were subcutaneous [12]. These subcutaneous tumours behave much less aggressively than the retroperitoneal tumours or those in the deep soft tissues [11,12]. Well differentiated liposarcomas have no known metastatic potential [14]. Metastasis would only be likely to occur after de-differentiation.

The management of this case can be compared to that of patients who have numerous unresectable neurofibromas with the risk of tumour progression to high grade malignancy.

The patient has been warned that there is a possibility of de-differentiation to a higher grade tumour. Patient self assessment and regular checks in the outpatient clinic are being carried out to identify firm or rapidly growing nodules or painful nodules if they develop.

### Conclusion

To summarise, germline retinoblastoma mutations in certain patients with hereditary retinoblastoma increase their susceptibility to developing benign tumours such as lipomas as well as malignant neoplasms, including soft tissue sarcomas such as liposarcoma. Patients with retinoblastoma and lipomas are even more at risk of developing further neoplasms. The possibility of either de-differentiation of the liposarcoma, or the development of a further primary malignancy are concerns for the future.

In conclusion, we describe a patient with extraordinary body habitus and a history of multiple fatty tumours, both benign and low grade malignant. He had a past history of bilateral retinoblastomas. The complex management issues related to this patient are manifest.

### References

1. Kaelin WG. Recent insights into the functions of the retinoblastoma susceptibility gene product. Cancer Invest 1997;15:243.

2. Dahlen A, Debiec-Rychter M, Pedeutour F, Domanski HA, Hogland Bauer HC, Rydholm A, Sciot R, Mandahl N, Mertens F. Clustering of deletions on chromosome 13 in benign and low-malignant lipomatous tumors. Int J Cancer 2003;103(5):616–623.
3. Scheffer H, Van Der Vlies P, Burton M. Two novel germline mutations of the retinoblastoma gene (RB1) that show incomplete penetrance, one splice site and one missense. J Med Genet 2000;37:E6.
4. Moll AC, Imhof SM, Bouter LM, Kuik DJ, Den Otter W, Bezemer PD, Koten JW, Tan KEWP. Second primary tumours in patients with hereditary retinoblastoma: A register-based follow-up study, 1945–1994. Int J Cancer 1996;67:515–519.
5. Eng C, Li F, Abramson DH, Ellsworth RM, Wong L, Goldman MB, Seddon J, Tarbell N, Boice JD. Mortality from second tumours among long-term survivors of retinoblastoma. J Natl Cancer Inst 1993;85: 1121–1128.
6. Li FP, Abramson DH, Tarone RE, Kleinerman RA, Fraumeni JF, Boice JD. Hereditory retinoblastoma, lipoma and second primary cancers. J Natl Cancer Inst 1997;89:834.
7. Abramson DH, Ellsworth RM, Kitchin D, Tung G. Second nonocular tumors in retinoblastoma survivors. Are they radiation-induced? Opthalmology 1984;91: 1351–1355.
8. Draper GJ, Sanders BM, Kingston JE. Second primary neoplasm in patients with retinoblastoma. Br J Cancer 1986;53:661–671.
9. Reider H, Lohman D, Poensgen B, Fritz B, Aslan M, Drohm D, Angersbach FJS, Rehder H. Loss of heterozygosity of the retinoblastoma (RB1) gene in lipomas from a retinoblastoma patient. J Natl Cancer Inst 1998;90:324–326.
10. Genuardi M, Klutz M, Devriendt K, Caruso D, Stirpe M, Lohmann DR. Multiple lipomas linked to an RB1 gene mutation in a large pedigree with low penetrance retinoblastoma. Eur J Hum Genet 2001:9;690–694.
11. Nascimento AG. Dedifferenciated liposarcoma. Semin Diagnost Pathol 2001;18:263–266.
12. Henricks WH, Chu YC, Goldblum JR, Weiss SW. Dedifferenciated liposarcoma. A clinicopathological analysis of 155 cases with a proposal for an expanded definition of dedifferenciation. Am J Surg Pathol 1997;21:271–281.
13. Lucus DR, Nascimento AG, Sanjay BKS, Rock M. Well differenciated liposarcoma. The Mayo Clinic experience with 58 cases. Anat Pathol 1994;102:677–683.
14. Evans HL, Soule EH, Winkelmann RK. Atypical lipoma, atypical intramuscular lipoma, and well differenciated retroperitoneal liposarcoma. A reappraisal of 30 cases formerly classified as well differentiated liposarcoma. Cancer 1979;43:574–584.

